# Dual Clinical Collaborator: A Pragmatic Role of nurses from developing countries**

**DOI:** 10.17533/udea.iee.v40n2e01

**Published:** 2022-09-15

**Authors:** Uma Ohri, Lakshmi Nirisha P, Vijayalakshmi Poreddi, Narayana Manjunatha, Naveen Kumar C, Suresh BadaMath

**Affiliations:** 1 M.Sc.(N), Nurse Informaticist. Telemedicine Center, Department of Psychiatry. National Institute of Mental Health and Neuro Sciences -Institute of National Importance-, Bangalore (India)Email: umaohri.11@gmail.com National Institute Of Mental Health And Neurosciences Telemedicine Center Department of Psychiatry National Institute of Mental Health and Neuro Sciences -Institute of National Importance- India umaohri.11@gmail.com; 2 MD in Psychiatry. Assistant Professor Department of Psychiatry. Email: nirisha1810@gmail.com National Institute of Mental Health and Neuro Sciences -Institute of National Importance-, Bangalore (India) National Institute Of Mental Health And Neurosciences Department of Psychiatry National Institute of Mental Health and Neuro Sciences -Institute of National Importance- Bangalore India nirisha1810@gmail.com; 3 Ph.D. College of Nursing, Email: pvijayalakshmireddy@gmail.com. Member of the Editorial Board of Investigación y Educación en Enfermería. National Institute of Mental Health and Neuro Sciences -Institute of National Importance-, Bangalore (India) National Institute Of Mental Health And Neurosciences National Institute of Mental Health and Neuro Sciences -Institute of National Importance- Bangalore India pvijayalakshmireddy@gmail.com; 4 MD in Psychiatry. Associate Professor, Department of Psychiatry. Email: manjunatha.adc@gmail.com National Institute of Mental Health and Neuro Sciences -Institute of National Importance-, Bangalore (India) National Institute Of Mental Health And Neurosciences Department of Psychiatry National Institute of Mental Health and Neuro Sciences -Institute of National Importance- Bangalore India manjunatha.adc@gmail.com; 5 MD in Psychiatry. Professor, Department of Psychiatry. Email: cnkumar1974@gmail.com National Institute of Mental Health and Neuro Sciences -Institute of National Importance-, Bangalore (India) National Institute Of Mental Health And Neurosciences Department of Psychiatry National Institute of Mental Health and Neuro Sciences -Institute of National Importance- Bangalore India cnkumar1974@gmail.com; 6 MD in Psychiatry, Professor. Department of Psychiatry. Email: nimhans@gmail.com National Institute of Mental Health and Neuro Sciences -Institute of National Importance-, Bangalore (India) National Institute Of Mental Health And Neurosciences Department of Psychiatry National Institute of Mental Health and Neuro Sciences -Institute of National Importance- Bangalore India nimhans@gmail.com

Collaboration is crucial in Professional nursing practice. Nurses act as a liaison between physicians and patients and their family members. Thus, it is vital to define the collaborative role of nurses in developing countries. The authors discuss pragmatic nurses’ role by adopting the Dual Clinical Collaborator model to ensure offering the quality of care to their clients. Nursing is a healthcare profession that focuses on the care of individuals and their families to help them recover from illness and maintain optimal health and quality of life.^1^ Health Care Professionals (HCPs) work together to provide quality health care and accomplish common goals. As healthcare delivery is becoming more complex, collaboration among healthcare workers and the patient can be a path to improve the quality of healthcare services. According to Walker and Avant's method, the conceptual definition of collaboration in nursing is an intra professional or interprofessional process by which nurses come together and form a team to solve patient care or healthcare system problem with members of the team respectfully sharing knowledge and resources.^2^ Thus, collaboration is crucial in everyday professional nursing practice and should be considered a core value of nursing.^3^

Contemporary nursing practice is based on the principles of person-centred care, shared decision-making and multidisciplinary teamwork.^3^. Nurses, being the first point of contact to their clients and offering direct care, play a unique role in collaborating with health care team members and patients as well as with family members. While there is a significant transition in the role of nurses from bedside care to Advanced Nurse Practitioners, the fundamental role in collaborating with physicians and patients and their families remains the same. However, most of countries are seeking to improve health care delivery by reviewing the roles of health professionals, including nurses.^4^ Therefore, this concept article aims to define the pragmatic role of nurses for better communication, research and, policy purposes. Also, this paper informs the international nursing fraternity about the key issues in nursing practice in India.

Globally, nurses’ roles are being greatly transformed to provide safe, ethically sound, patient-centered care.^5^ However, much of this transformation has occurred in developed countries such as the United States, the United Kingdom, Canada, and Australia.^6^ Nurses in developed countries are recognized as independent professionals with additional training and qualifications. Nurses can perform health assessments, and plan and implement nursing care interventions independently. The various roles of nurses include registered nurses, specialty nurses, advanced practice nurses, clinical nurse practitioners, nurse researchers, nurse educators, Health and Welness counsellors etc. The reason for developing more advanced functions for nurses in these countries was to improve access to care, promote a higher quality of care, and provide more intensive follow-up and counseling for patients with chronic illness in primary care. When the quality of care is improved, it leads to fewer complications, reduced healthcare expenditure, and prevents unnecessary hospitalizations. Another reason for these advanced roles is to enhance nurses' retention rates and enhance their career prospects.

Nurses in developing countries either work as clinical nurses in various settings or as nurse educators. The nurse practitioner role has not been formally recognized in most of the developing countries such as in India. However, initiatives by the Indian Nursing Council (INC) are underway to implement education programs for a formal nurse practitioner role.^7^,^8^ The role of nurses in India revolves around clinical and supervisory positions. Their role is seen chiefly as bedside care and medication administration. As the nurses gain experience over the years, the function might get shifted to a supervisory position. In a typical Indian hospital setting, we would observe a nurse doing various tasks related to the patient. The nurses provide care, maintain records and reports related to the patients. Many non-nursing roles are performed by nurses, such as billing, record keeping, inventory, laundry, diet, etc., which also diverges from their fundamental role, which is patient care.^9^ The role of nurses is considered to provide medications to patients and manage the hospital wards, and their role becomes stagnant over time as there are significantly fewer growth opportunities.^10^ There is a lack of leadership roles and decision making of nurses in the treatment of patients. In fact, in India, patients and families are more comfortable with nurses than doctors, which might be possible because nurses spend more time with patients than doctors. Patients are under the supervision of nurses during their time in the hospital. Hence, the role of nurses needs to be defined pragmatically.

In this article, we would like to discuss how a nurse collaborates concurrently with the patient and the doctor to maintain the first contact care and continuity of care. We want to name this collaboration "Dual Clinical Collaboration (DCC) and the role of nurses as Dual Clinical Collaborator." It involves collaboration between the nurse, patient, and doctor, and the nurse is the center of this collaboration. The prerequisites for any partnership to work are common goals, open communication, and mutual respect. The foremost part of it involves how the nurse receives the information from the patient, how they share this information with the doctor and the efficiency of using it for planning patient care.

During a hospital visit/stay, a patient interacts with different employees, including Doctors, nurses, and other HCPs. It is essential to make these interactions smooth and straightforward for patients and their family members. Nurses carry the influence and have the opportunity to play this role effectively as patients spend most of their time under their care and supervision. A nurse builds a trusting relationship with the patient, which helps to understand the patient’s values and beliefs. It becomes easy for the nurse to plan the care when there is clarity about the patient's expectations from the health care providers. How effectively a patient can express his concerns depends on how well the nurse receives the information shared by the nurse. Effective collaboration with patients allows the patient and family to participate actively in the treatment process and improves their experience. 

Historically, general practice has concentrated on doctors providing care; it is now necessary that we recognize how doctors and nurses can cohesively offer high-quality care. Seamless collaboration between nurses and doctors is essential for effective and efficient health care delivery. The doctor-nurse relationship, which was traditionally considered hierarchical, is now evolving into a collaborative relationship. Communication between nurses and physicians is a critical part of the information flow in healthcare. Collaboration between physicians and nurses means cooperation in work, sharing responsibilities, and making decisions to formulate and carry out plans for patient care. A collaboration between the nurse, patient, and the doctor would only be effective if the doctor and the nurse have a good communication and awareness of each other’s roles and responsibilities. Communication between the doctor and the nurse is necessary given the interdependence of the two professions and their primary role in safe and quality patient care. It can improve patient outcomes, lower healthcare costs, increase job satisfaction, and maintain patient safety.^11^ Meanwhile, the growing evidence shows that improper or poor communication can create a chronic state of conflict between nurses and physicians, leading to increased medical errors and poor outcomes. 12,13

Dual Clinical Collaboration (DCC) helps take advantage of the nurse and doctors' knowledge and experience, familiarizing themselves with each other's skills and perspectives and leading to professional development. The workload is shared, and it also leads to job satisfaction. It can also be a way to enhance leadership and decision-making in nursing. A nurse decides what kind of care their patients require and then collaborates with the health team accordingly.

## Conclusion

Health care is dynamic; the demands of healthcare rapidly increase and require an extra workforce. While we face a shortage of healthcare workforce, we need to be innovative in utilizing the available resources. The concept of nurses collaborating with the doctor and the patient is not new, and it is something that is done daily without realization. However, it has not received due attention and needs further exploration. It provides nurses with an opportunity for professional advancement and refining their skills through clinical learning. DCC makes health services easily accessible, untroublesome to patients, helps understand their individual needs and strengthens the overall health care system. Our primary institutions can work on the curriculum such that it involves DCC, which could make it easier for future professionals to adapt to this concept swiftly.

## References

[1] Yolanda S. (2019). Nursing Healthcare Profession.. News Med. Life Sci..

[2] Emich C. (2018). Conceptualizing collaboration in nursing. Nurs.

[3] Wiltjer H. (2017). Why collaboration should count as a core value of nursing. Nurs. Times.

[4] Delamaire M-L, Lafortune G. (2010). Nurses in Advanced Roles. A Description and Evaluation of Experiences in 12 Developed Countries. OECD Health Working Papers.

[5] Salmond SW, Echevarria M. (2017). Healthcare Transformation and Changing Roles for Nursing. Orthop. Nurs.

[6] Younas A. (2020). A Comparison of Nursing Practice in Pakistan with International Nursing Practice. Creat. Nurs..

[7] INC. Indian Nursing Council. (2016). Syllabus & Regulations; Nurse practitioner in critical care post graduate residency program..

[8] Putturaj M, Prashanth NS. (2017). Enhancing the autonomy of Indian nurses. Indian J. Med. Ethics.

[9] Chhugani M, James MM. (2017). Challenges faced by nurses in india-the major workforce of the healthcare system. Nurs. Care Open Acces J..

[10] Sharma SK, Kalpana T, Pastin PRP. (2020). Status of nurses in India: Current situation analysis and strategies to improve. J. Med. Evid.

[11] Reeves S, Pelone F, Harrison R, Goldman J, Zwarenstein M. (2017). Interprofessional collaboration to improve professional practice and healthcare outcomes. Cochrane Database Syst.

[12] Cypress BS. (2011). Exploring the concept of nurse-physician communication within the context of health care outcomes using the evolutionary method of concept analysis. Dimens Crit. Care Nurs.

[13] Tjia J, Mazor KM, Field T, Meterko V, Spenard A, Gurwitz JH. (2009). Nurse-physician communication in the long-term care setting: perceived barriers and impact on patient safety. J. Patient. Saf.

